# Novel decomposition of polycarbonate and effect for marine ecosystem†

**DOI:** 10.1039/d3ra04127a

**Published:** 2023-10-12

**Authors:** Koshiro Koizumi, Akifumi Okabe, Hideki Kimukai, Hideto Sato, Hiroyuki Taguchi, Masahiko Nishimura, Bum Gun Kwon, Katsuhiko Saido

**Affiliations:** a Collage of Science and Technology, Nihon University Funabashi Chiba Japan koizumi.koushirou@nihon-u.ac.jp; b Albatross Alliance Tokyo Japan; c Department of Architecture and Living Design, Junior College of Nihon University Funabashi Chiba Japan; d School of Pharmacy, Nihon University Funabashi Chiba Japan; e Atmosphere and Ocean Research Institute, The University of Tokyo Kashiwa Chiba Japan; f Chosun College of Science & Technology Gwanju South Korea

## Abstract

Analysis of pollution of the ocean plastics is presently being extensively carried out but special attention should be direct to matters. It is widely believed that plastic dose not decompose in the ocean. Certain contaminants, bisphenol-A (BPA) that serves the material for polycarbonate (PC) and epoxy resin (EPX) both of which may possibly be elute or degrade from commercial products, have often been detected in rivers, lakes and oceans. To clarify in detail the extend of this impact of this situation, purified PC (BPA free) was decomposed at temperatures range 50–230 °C. PC was seen to start decomposing at 50 °C over a 3 day period to generated 11 μg kg^−1^ BPA. Based on the rate constants of BPA, the activation energy was calculated 42.0 kJ mol^−1^. Since this value is almost same as the EPX and polystyrene (PS) of each decomposition. Based on the PC decomposition rate and the actual BPA value in the deep sea, the 280 million metric tons (MT) BPA in the world ocean was estimated. Unlike plastics, BPA shows endocrine disrupting in fish. It should thus be considered that degraded PC and EPX pose a serious threat to the marine ecosystem, directly.

## Abstract 2

Drifting plastics not only crushed into micro/nano-particles, but also PC and EPX generate monomer/BPA, BPA revealed a direct affect on the marine ecosystem.

## Graphical abstract

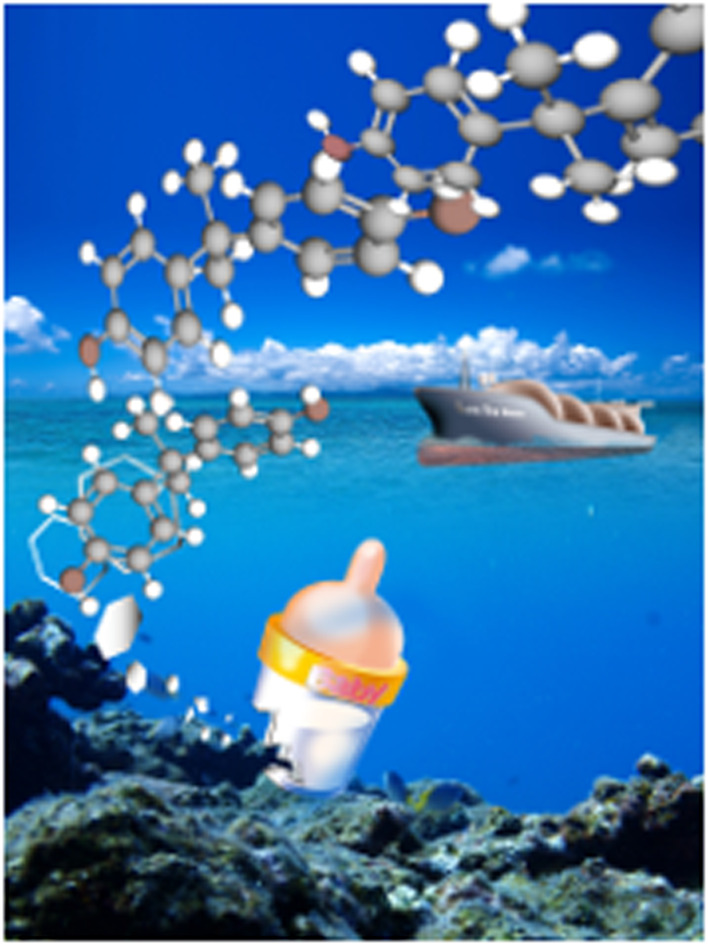

## 1 Introduction

Whether accidentally or intentionally, waste plastic from land sources ultimately makes its way into world oceans.^1–3^ Since 1972, lumps of plastic breakage into micro/nano-pieces have been clearly shown a serious and large source of ocean pollution.^4–6^ Drifting plastics were reported accidental entanglements for marine animals, and caused the miss ingestion such as macro to micro/nano-particles for marine biota of whale to plankton has been reported.^7–10^

Since the late 1980s, BPA contaminations have been detected in rivers, lakes and oceans.^11–14^ BPA [2,2-bis(4-hydroxyphenyl)propane] is material for PC and EPX, commercial production started in 1957 USA. In 2020, the estimated production of PC in USA was approximately one million metric tons (MT).^15^ PC has a wide range of application such as heat-resistant, light weight with high durability, transparent, impact resistant and shows flame retardancy. All these qualities are well proportioned. The reasons why PC is developing and is finding new innovative uses after another so that its production is yearly 4% increasing.^16^ The authors investigated styrene oligomers (SOs)‡‡SOs [styrene oligomer: SOs means the mixture with the constant ratio of styrene(monomer) 1: 2,4-diphenyl-1-butene (styrene dimer) 1: and 2,4,6-triphenyl-1-hexene (styrene trimer) 5]. contaminations in sea water and sand more than 25 countries in the Northern Hemisphere costal area and surveyed at open ocean water surface to 5000 m depth in Northwest Pacific Ocean.^17–20^ SOs and BPA were detected at all survey sites. BPA along the Pacific coast was found to exceed SOs and shown some hot spots.^11^ The source and elution pathways of BPA in world oceans have yet to clearly elucidated.^21^ Numerous studies have been conducted to assess the chemical stability of plastics under conditions of ageing and exposure to weather.^22–25^ However, to date, no kinetic research on PC decomposition has been conducted at the lower temperature range in the natural environment.^26–30^

Using purified PC, novel decomposition at low temperature 50–230 °C, was thus examined with polyethylene glycol (PEG) as a heating medium. Based on generated BPA from 50 to 200 °C, the rate constants of PC were determined at each temperature range. The kinetic parameters of the PC decomposition were determined and the activation energy of the conversion of PC into BPA was given as 42.0 kJ mol^−1^. Since this value is almost same as the EPX (42.0 kJ mol^−1^)^29^ and PS (47.0 kJ mol^−1^).^17^ Based on kinetic data,^17,27^ one MT of PC was found to decompose at a rate of 0.3 g per year at 30 °C. Simulation^27^ indicated that the total amount of degraded PC in the ocean has been as much as 450 MT during the period, 1950 to 2050. Drifting PC not only breaks up into micro/nano sized particles, but subsequently degrades into BPA. It should thus be considered that degraded PC and EPX in the oceans pose a direct serious threat to the marine ecosystem.

## 2 Experimental

### 2.1 Materials and methods

#### 2.1.1 Reagent

Polyethylene glycol (PEG1540, average molecular weight 1350 to 1650, Wako Pure Chemical Co.) was used. Dichloromethane (DCM) and diethyl ether (DE) for dissolving PC or reprecipitation and tetrahydrofuran (THF) as the GPC eluent, all these were of reagent grades and manufactured by Wako Pure Chemical Co. *N*,*o*-Bis(trimethylsilyl)trifluoroacetamide (BSTFA) were used of Supelco Co. Ltd. The BPA standard for calibration is used of reagent grade by Aldrich. The internal standard, phenanthrene (PH) and surrogate, biphenyl (BP) was used after being purified using a reagent grade of Kanto Chemical Co., Inc. following sublimation treatment.

#### 2.1.2 Preparation of reaction material

**Fig. 1:**
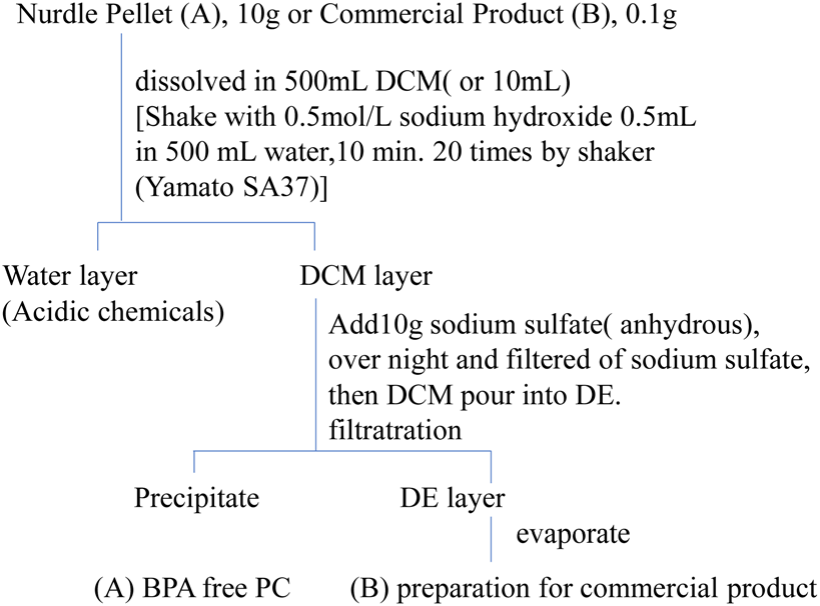
Preparation for reaction material and commercial samples.

**Fig. 2:**
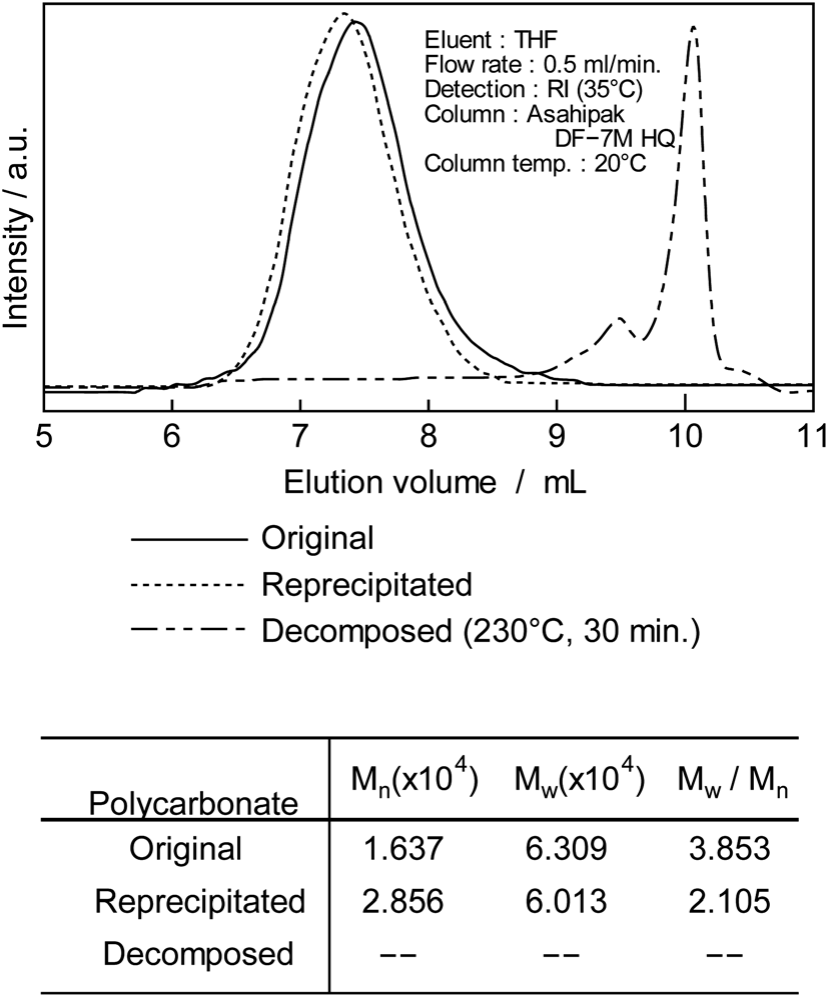
GPC chromatogram of original, purified and decomposed PC (230 °C, 30 min).

PC nurdle (virgin pellet) and products contained BPA which caused unreacted BPA or generated from heat treatment of production. In this study, PC nurdle pellets (Panlite/Teijinkasei Co. Ltd Tokyo Japan) from a supplier contained 200 mg kg^−1^ of BPA and some additives.^31,32^ 10 g nurdle PC solved in 500 mL DCM and add 0.5 mL, 1 mmol sodium hydroxide with 500 mL water. The solution was shaken for 20 min by Yamato shaker SA-31 for ten times. The DCM layer is recovered and add 10 g sodium sulfate (anhydrous) overnight. After filtrations, the DCM solution is poured into 2 L DE and purified PC (BPA-free) is precipitated. Details were shown in Fig. 1. This BPA-free PC (*M*_n_: 28 600, Fig. 2) was used after 10 days drying in vacuum (3 mm torr) at 25 °C.

### 2.2 Decomposition method

The silicone oil bath was placed on stirrer bearing with a heat regulator. The thermal decomposition of PC was carried out in 4.9 g of PEG in a 30 ml round-bottomed flask bearing with Y-tube and stirrer chip on the oil bath. The Y-tube was attached with a thermocouple, an introduction tube of N_2_ gas and reflux condenser, respectively. N_2_ gas of which was adjusted 50 ml min^−1^ by the regulator from a N_2_ cylinder. When the internal temperature reached the fixed temperature, 0.100 g of purified PC was added to the medium with mixing by stirring at 500 rpm. The reaction temperature was adjusted and retained ±1 °C of the fixed temperature by thermocouple and digital thermometer (CUSTOM CT-1310) inserted in the reaction solution directly. The detail of the method was shown previously.^11,17,29,30^

### 2.3 Preparation for analysis of reaction mixture

After reaction at the fixed temperature for the fixed time, the reaction mixture dissolved in 10 ml DCM and recovered was transferred to a 300 mL separatory funnel was washed with 50 ml water to remove the PEG. 2 g anhydrate sodium sulfate was added into the DCM layer overnight. After filtrate, the DCM solution was poured into 10 mL DE to precipitate PC/polymer part. The DCM/DE solution was filtrate and drying using a rotary evaporator, evaporated under 30 °C. As a residue an internal standard (PH) was added to 0.5 mg kg^−1^ used as a sample for GC/MS measurement. The GC/MS sample was prepared by removing the polymer by the method shown in Fig. 1A and B.

### 2.4 GC-MS analysis

1 μL of samples was separately injected into a 6890 gas chromatograph (Agilent Technologies, Avondale, PA, USA) coupled to a Jeol JMS-AII electron impact ionization mass spectrometer. The analysis was performed in the splitless mode into a DB-5 capillary column (30 m × 0.32 mm I.D., 0.25 μm film thick, Agilent Technologies, Avondale, PA, USA). The oven temperature for the analysis was programmed from 50 °C (hold 2 min) to 290 °C at 15 °C min^−1^ and then where it was maintained for 5 min. Helium gas was used as a carrier gas at constant flow of 1.2 mL min^−1^. The MS transfer line temperature was maintained at 300 °C, and the ion source and quadrupole at 210 °C and 255 °C, respectively. Measurements were performed in SCAN (50–400 *m*/*z*, 300 ms) and SIM (selection monitoring) modes and the *m*/*z* followed are listed in Table 1. Field survey samples were predicted to have low BPA. Therefore, the sample was treated with BSTFA to increase its volatility (TMS derivatives).

**Table 1:** Mass spectrometry apparatus conditions

Apparatus column	MS: Jeol LMS-AM II and GC: HP-6890 DB-1 (*L*: 30 m, ID: 0.32 mm, film thick: 0.25 μm)		
**Monitoring ion**			
TIM: all *m*/*z*, scan 300 ms, 5:10 to 21:00 min		Column temperature	50° (2 min hold) upto 290 °C
SIM: *m*/*z*: 76, 135, 150, 154, 213, 228, 325, 340		(Program rate: 15 °C min^−1^ and 5 min hold at 290 °C)	
Injection system	Split less	Interface temperature	255 °C
Injection	Manual, 1 μL	Ion source temperature	210 °C
Injection temperature	250 °C	Ion acceleration current	70 eV
Purge time	3 min	Current	300 μV
Purge flow	30.0 mL min^−1^	PM voltage	600 V
Total flow	33.7 mL min^−1^	Carrier gas	He (flow: 1.4 mL min^−1^)
Save flow	15.0 mL min^−1^		
Save time	15.0 s		

### 2.5 Preparation of a calibration

Preparation of a calibration curve using standard substances for determination 0.1 g 10 mL^−1^ of the standard stock solution was prepared and diluted to prepare solutions of 0.01, 0.05, 0.1, 1, 3, 5, 10, 20, 50 mg kg^−1^ 10 mL^−1^, and 1 μL thereof was injected into GC/MS. By analyzing the mass spectrum of each standard substance obtained by GC/MS scan mode measurement (TIM) and grasping the *m*/*z* of the main fragment ion, the monitoring channel of the substance to be measured in selective ion monitor (SIM).

### 2.6 Apparatus

The quantitative determination of decomposition products and field survey was conducted using GC/MS. The following apparatus was used for the separation and identification of the products; nuclear magnetic resonance spectroscopy (NMR): Jeol LNM-LA500 FT NMR system (^1^H and ^13^C), gas chromatography-mass spectrometer (GC/MS), GC: HP6890 and MS: Jeol JMS-AMII quadrupole mass spectrometer, gel permeation chromatography kai Co. Ltd. Detector, Shodex RI-71, temp: 35 °C, (column: Asahipak GF-7M HQ, Asahi Chem. Ind. Co., Ltd temp: 20 °C). High-mass spectrometer: Hitachi M-2000 (double-focusing mass), Infrared spectroscopy (IR): JASCO FT-IR300E.

## 3 Results and discussion

### 3.1 Accuracy of analysis

Qualitative analysis was performed by total ion monitor (TIM) and quantification was performed by SIM in this study. The conditions of GC/MS were shown in Table 1. In SIM, choosing the monitor ion (qualitative ion Q: *m*/*z* 213 and identification ion I: *m*/*z* 228 or TMS derivatives, Q: *m*/*z* 357, I: *m*/*z* 372), the calibration curve of each target chemical was obtained using internal standard PH and the BPA standard solution. The correlation coefficient (*r*) was obtained based on linearity within 0.01–10 mg kg^−1^, *R* = 0.9996–0.9999. The required detection limit was determined as 5 μg kg^−1^, in S/N = 2. The relative standard deviation of peak area of the PH for each injection was within 2%. The eliminate error for extraction recovery of surrogate DP was found to range from exceed over 90.3–97.8%, with an overall average of 95.2%, and relative standard deviation (RSD), was always within 7.3%.

### 3.2 Analysis of commercial products

Brotons,^33^ Howe,^34^ and Romero^35^ canned foods obtained from different countries and detected BPA in all samples. But the reasons as to why BPA is present in canned food remain to be determined.^21,36^ Especially, canned food or nursing bottles are heat at about 100–120 °C for several minutes each time for sterilization. The commercially available PC product, (nursing bottle, food container) was cut with scissors, exactly 0.100 g was weighed by an analytical balance, dissolved in 5 ml of DCM, and BP was added as a surrogate so as to be 0.1 mg kg^−1^ at the time of measurement as shown. This solution was added to 10 ml of DE to precipitate and remove the PC-polymer portion. The DE solution was concentrated using a rotary evaporator until immediately before drying. Details were shown in Fig. 1B.

To this, PH as an internal standard was added so as to be 0.5 mg kg^−1^ at the time of measurement to make exactly 10 ml acetone solution. 1 μL of this solution was injected into a GC/MS and separated and quantified by SIM. The commercial products in Table 2 are made by the injection molding. In this method, the nurdle PC pellets are extruded by a screw to 200–250 °C after being dried with air and put into a mold. The mold is heated to 120–150 °C, where a PC is introduced and molded into the desired shape. After cooling, the mold is cooled, and the product is peeled off from the mold and collected. The mold, preheating temperature, time and additives^31^ for improving durability during this process are not clear as know-how from manufacturers to molding processing companies. BPA was found present in all commercial products^28^ to 111 mg kg^−1^ as shown in Table 2.

**Table 2:** Analytical results of commercial food containers

Sample	1	2	3	4	5	6	7	8	9	10	11	12	13
Component	Milkbottle								Cup	Bottle	Container	Coffee dripper	Milkbottle
	USA		Germany	Italy	France	Japan							China
BOL	ND	ND	9.20	ND	ND	17.50	ND	ND	2.30	ND	ND	12.80	ND
BPA	111.07	38.13	52.07	88.86	45.46	50.69	46.00	42.43	47.13	47.34	28.27	102.49	94.11
	BOL: *t*-butylphenol								(Unit; μg kg^−1^)				
	BPA: bisphenol A												

Differences in BPA contain were due to these in heat treatment conditions, temperature and time for the molding and processing of each product, and the synthetic method for nurdle pellet. These BPA would thus appear to be exuded from the PC-polymer matrix temperature/time-dependently into the ocean.

### 3.3 Temperature effects for PC decomposition

After treating DCM/DE, purified PC was prepared as shown in Fig. 1A. Purified PC for thermal decomposition with a BPA of 5 μg kg^−1^ or less was prepared due to sodium hydroxide and reprecipitation. Average molecular weight (*M*_n_) of PC was determined as 28 600 from the GPC retention time (*T*_R_: 13–17 min) and convention calibration curve as shown in Fig. 2, solid original line. The solid line is the original nurdle PC and the dashed line is the purified (BPA free) PC and double dashed line is decomposed PC at 230 °C, 30 min. The PC molecular is almost same before and after purification of PC sample as shown in Fig. 2.

Thermogravimetric (TG) and differential scanning calorimetry (DSC) analysis showed that the nurdle PC had a glass transition of 130 °C and the purified PC of 160 °C. All significant weight reductions in TG were over 350 °C, respectively.

After decomposition, there were no PC peak but low molecular chemicals were newly appeared the retention time at 10 min.

**Fig. 3:**
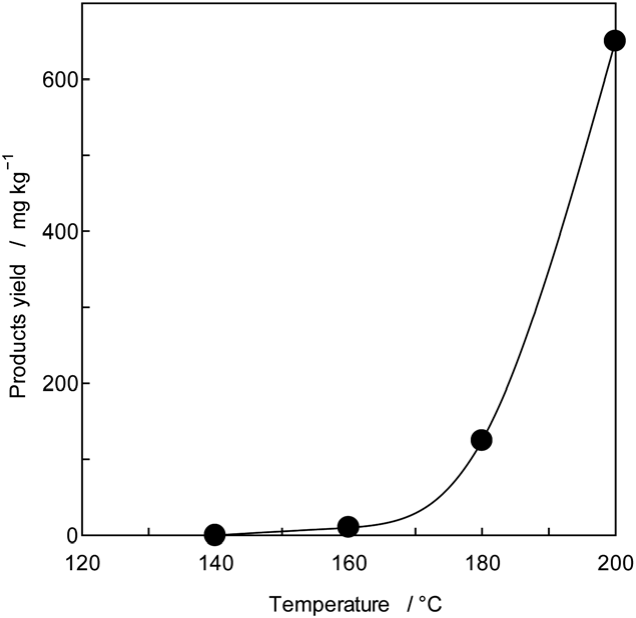
Effects of reaction temperature (time: 60 min).

To confirm the generation of BPA from PC in the oceans, using purified PC as the reactant, a novel thermal decomposition was examined with PEG.^17,29,30^ BPA was extracted from reaction mixtures for liquid/liquid distribution system with water and DE to remove unreacted PC and PEG as shown in Fig. 1. The quantities of BPA were measured by SIM-GC/MS. The results are presented in Fig. 3 and Table 3. It has been said that PC does not decompose below 250 °C.^28^ However, this study kinetically revealed that PC is completely decomposed at 230 °C, 30 min as shown in Fig. 2 double dot line. Fig. 3 shows the effect of temperatures at fixed time (time: 60 min). Table 3 shows the rate constant and temperature in absolute temperature at each reaction, calculated based on the actually measured BPA from the extension of reaction time at each temperature on PC decomposition. In Fig. 3, the amount of BPA was small at 50 °C, it could not be expressed on the same axis. Therefore, the velocity constant under 150 °C were shown in Table 3. BPA was noted to increase with rise in temperature rectilinearly at 160 °C in Fig. 3. Main PC decomposition product was BPA and *t*-butylphenol. Trace amounts of phenol, cresol, *t*-butylphenol, *t*-butylhydroxy toluene, diphenylcarbonate and 5-cumyl phenol were qualified at high temperature range (160 °C or more). After 120 °C, 120 min decomposition, new signals were measured in ^1^H-NMR at 3.50 to 3.79 ppm and ^13^C-NMR at 61.620, 70.189, 70.502 ppm, respectively. These new signals were attributed to alkanes and alkenes by scission of carbonate or isopropylidene – groups.^37,38^

**Table 3:** BPA rate constant at each reaction temperature

Reaction temp.		1/*T*	BPA rate constants	
°C	K		*k*	ln *k*
50	323.15	0.003095	0.00279	−5.88171
80	353.15	0.002832	0.01311	−4.33510
120	393.15	0.002544	0.0555	−2.89137
150	423.15	0.002363	0.1587	−1.84074
180	453.15	0.002207	0.3174	−1.14759
200	473.15	0.002113	0.4340	−0.83471

In the IR spectrum, new absorption of 3430 (–OH), 2874 (CH_3_–C–CH_3_), 1644 (–CO–) and 1193 (–CO–) cm^−1^ attributed to BPA was observed.^22,24^ The result indicates that PC main chain scission may occur in this condition. *t*-Butylphenol as a polymerization inhibitor was detected at each condition. But the formation of this chemical showed no correlation with reaction temperature.

### 3.4 Effect of time and calculation of activation energy

**Fig. 4:**
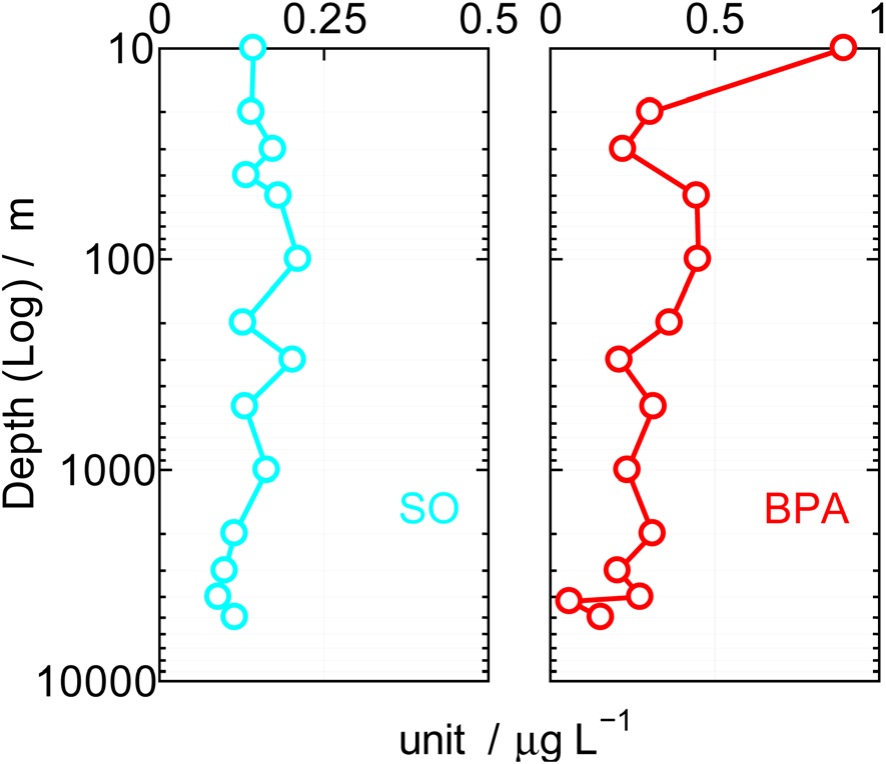
Vertical BPA and SO value in Northwest Pacific Ocean.

BPA increases good linearly with the extension of the reaction time at 50–200 °C as shown in Fig. 4. Assuming that the thermal decomposition of purified PC was the zero-order reaction,^29,30^ the rate constants of the BPA were determined at a temperature range 50–200 °C. The results were shown in Table 3.

Based on Table 3, Arrhenius-plots were made for the reciprocal of absolute temperature (*k*^−1^) and ln *k*. The logarithmic of each PC rate constant (ln *k*) showed good linearity (*R*^2^ = 0.997) with the reciprocal of absolute temperature (1/*T*, *k*^−1^). The activation energy of PC from BPA generation was found to be 42.0 kJ mol^−1^, thus confirming the kinetic data. Thus, PC is clearly shown not to be stable with heat application. Since this value is almost same as the EPX (42.0 kJ mol^−1^)^29^ and PS (47.0 kJ mol^−1^)^30^ of each decomposition.

### 3.5 Field survey and simulation of BPA

The coastal region BPA in water and sand were affected by the latitude, longitude meteorology, ocean currents, weather,^23,24^ and population density. Using the Japanese Government research ship Hakuho along the 10° to 45°N latitude, 133° to 160° longitude, 5000 km north to south and 2000 km east to west, during the cruise (B: KT10-25, 31st October to 17th November 2010. C: KH12-3, 6th July to14th August 2012) in the Northwest Pacific Ocean. Distributions of SOs and BPA were surveyed from surface to bottom (5000 m) with 10 L of water at a fixed depth each (Niskin water sampler/General Oceanics, Inc.).

The results were shown in Table 4 and Fig. 4. Field survey predicted very low BPA. In addition, since BPA is highly adsorbable to the GC column, it was analyzed by TMS derivative. In Fig. 4, the depth of the sea is shown logarithmically on the vertical axis (left/m), and the SOs and BPA value detected at each depth is shown μg L^−1^ on the horizontal axis. The mean surface current was 0.7 knots during this cruise. SOs and BPA tend to be high in the photic zone at a depth of 200 m in Fig. 4, but at more than 2000 m where there is only minimal horizontally currents and therefore more limited water mixing, means SOs and BPA levels approximated 0.1 to 0.2 μg L^−1^, respectively. The SOs and BPA both would thus appear to adhere or be adsorbed by organic or inorganic materials and this flocculation to particulate matter may be a mechanism for its vertical movement.

**Table 4:** Details of BPA at Northwest Pacific Ocean (unit: μg L^−1^)

Depth (m)	B6	B9	B13	C1	C5	C9	C12	C13	C14	C15	C16	C17	C18
10	0.414	0.576	0.219	0.084	0.039	1.497	—	2.321	—	0.192	1.330	0.100	0.071
20	0.442	0.554	0.200	0.161	0.038	0.070	—	1.406	0.074	0.108	0.105	0.090	0.410
30	0.397	0.504	0.202	0.150	0.062	0.072	—	—	—	0.321	—	0.066	0.220
40	—	—	—	—	—	—	—	—	0.050	—	—	—	—
50	0.421	0.577	0.147	0.103	0.137	0.054	—	1.262		1.869	0.115	0.055	0.165
75	—	—	0.170	—	—	—	—	—	—	—	—	—	—
100	—	1.269	0.476	0.145	0.063	0.212	—	1.590	0.116	0.249	0.073	0.526	0.234
200	0.408	0.568	0.164	0.426	0.051	0.082	—	0.194	—	1.668	0.231	0.171	0.039
300	—	—	—	0.656	0.152	0.112	—	—	—	0.158	—	0.144	0.044
500	0.314	0.302	0.090	0.132	0.107	0.056	—	0.396	0.070	0.956	1.071	0.204	0.043
1000	—	0.152	0.142	0.316	0.097	0.156	—	0.548	0.094	0.118	0.176	0.552	—
2000	0.393	0.162	—	0.159	0.059	0.474	—	0.229	—	0.613	0.406	—	—
3000	0.462	0.100	—	0.520	0.044	0.226	0.074	0.071	—	0.184	0.169	—	—
4000	0.656	0.168	—	0.241	0.043	0.277	0.071	0.713	—	0.159	0.147	—	—
5000	—	—	—	0.210	—	—	0.088	0.167	—	—	—	—	—
Average	0.420	0.448	0.221	0.280	0.074	0.244	0.232	0.789	0.081	0.550	0.651	0.268	0.230
Total	0.359												

The amounts of waste PC/EPX inflow accumulated in ocean bodies from 1950 to 2020 have been estimated as 1.5 × 10^7^ MT,^17^ assuming PC/EPX to constitute 7% of plastic global total production^16^ (4.8 × 10^8^ MT), and 3% of plastics^4^ to flow into world ocean. Using open ocean deep sea BPA values of 0.2 μg L^−1^ and total water in oceans' as 1.4 × 10^21^ L, as with PS,^17,30^ it is estimated that 280 million MT of BPA are present between 1950 and 2020.

## 4 Conclusions

It is widely believed that plastic does not decompose in the ocean. The results indicated the typical heat-resistant plastic PC to have little stability toward heat, kinetically. In all thermoplastics of polyethylene (PE), polypropylene (PP), polyvinyl chloride (PVC) and PS production, PC and EPX production is less than 6% of world plastic. World coastal survey indicated most of the drifting plastic to be composed PE, PP, PVC and PS. There are no PC nursing bottles or food container could be found all survey site in the world. But BPA (0.2 μg L^−1^) deeper than 2000 m in the Northwest Pacific Ocean are twice that of SOs (0.1 μg L^−1^) and higher than those derived from PS. The PC/EPX decomposition rate of BPA, activation energy was found to be 42.0 kJ mol^−1^. With the deep-sea BPA, it is estimated that there are 280 million MT of BPA in the world ocean. Surveys in coastal areas around the world have found few washed-up PC products. BPA also has a history of being widely used for thermal paper and a stabilizer for other plastics. On the other hand, EPX had the same activation energy as PC. It was suggested that EPX contributes to the supply of BPA in the ocean because it has been used as a ship hull paint for ocean voyage. Drifting macro plastics are thus shown not only to be crushed into micro-nano fragments, but also to degrade into BPA of which PC/EPX are made. In oceans in which PC or EPX has been discarded, there is the possibility of long-term pollution by BPA, generated *via* the decomposition of these plastics since tropical and subtropical area beach may remain at 50 °C or more for many years. Contamination by BPA in oceans is progressive since its amount increases with time and temperature. It should thus be considered that degraded PC and EPX pose a new and serious threat to the marine ecosystem, directly.

## Ethical statement

This article does not involve any human or animal subjects.

## Author contributions

Koshiro Koizumi: field survey, analysis and writing – original draft. Akifumi Okabe: field survey and calculation. Hideki Kimukai: field survey, analysis and editing. Hideto Sato: field survey (voage) and analysis, Hiroyuki Taguchi: PC decomposition, rate calculation and GPC analysis. Masahiko Nishimura: field survey and analysis. Bum-Gun Kwon: field survey and analysis. Katsuhiko Saido: field survey, decomposition, GC/MS analysis, writing & supervision.

## Conflicts of interest

There are no conflicts to declare.

## Supplementary Material

RA-013-D3RA04127A-s001
