# Supplementary material for: Novel decomposition of polycarbonate and effect for marine ecosystem
Source: RSC Adv. 2023 Oct 12;13(42):29668–74. doi: 10.1039/d3ra04127a (PMC10568682; doi:10.1039/d3ra04127a)
Supplement: RA-013-D3RA04127A-s001 [file RA-013-D3RA04127A-s001.pdf]

Electronic Supplementary Material (ESI) for RSC Advances.
This journal is © The Royal Society of Chemistry 2023

Supplimentary infromation

Acronyms and Abbreviations

BP	biphenyl	
BPA	bisphenol A	2,2-bis(4-hydroxyphenyl) propane
BSTFA		*N*, *o*-bis(trimethylsilyl)trifluoroacetamide
DCM	dichloromethane	
DDT	dichlorodiphenyltrichloroethane	
EPX	epoxy resin	
MT	metric tone	
PAE	phthalic acid ester	
PC	polycarbonate	
PCB	polychlorinated biphenyl	
PET	polyethylene terephthalate	
PH	phenanthrene	
POP	persist organic pollutant	
PS	polystyrene	
PVC	polyvinylchloride	
SD_1	styrene dimer	1,3-diphenyl-1-butene
SD_2	styrene dimer	2,4-diphenyl-1-butene
SIM	selected ion monitor	
SM	styrene monomer	
SOs	styrene oligomer	
ST	styrene trimer	2,4,6-triphenyl-1-hexene
TIM	total ion monitor	
